# The Overlooked Agent: Cytomegalovirus Colitis in an Immunocompetent Patient

**DOI:** 10.7759/cureus.36926

**Published:** 2023-03-30

**Authors:** Margarida Lagarto, Ana Santos, Bruno D Freitas, Marta Anastácio, Susana Jesus

**Affiliations:** 1 Medical Oncology, Centro Hospitalar de Lisboa Ocidental, Lisboa, PRT; 2 Internal Medicine, Centro Hospitalar de Lisboa Ocidental, Lisboa, PRT

**Keywords:** elderly population, persistent diarrhea, infectious colitis, immunocompetent patients, cytomegalovirus colitis

## Abstract

Cytomegalovirus (CMV) colitis is usually associated with immunosuppressed patients, which by the classic definition are individuals who have immunosuppressed associated conditions (human immunodeficiency virus [HIV], oncology diseases, inflammatory bowel disease, transplant patients) or who are submitted to immunosuppressing therapies (for instance, corticosteroids, chemotherapeutic agents or immunomodulation therapies). In immunocompetent patients, this diagnosis tends to be often missed, leading to a delay in initiating proper management. We present a case of a 91-year-old woman that was diagnosed with CMV colitis without any identified formal immunocompromising factors.

We intend to highlight the need to review the definition of an immunosuppressed individual and emphasize that CMV colitis should be considered in the differential diagnosis, especially in elderly patients and those with underlying conditions that can possibly affect their immune status, since prompt diagnosis and treatment are essential and influence the prognosis.

## Introduction

Cytomegalovirus (CMV) colitis is usually associated with immunosuppressed patients, which by the classic definition are individuals who have immunosuppressed associated conditions (human immunodeficiency virus [HIV], oncology diseases, inflammatory bowel disease, transplant patients) or who are submitted to immunosuppressing therapies (for instance, corticosteroids, chemotherapeutic agents or immunomodulation therapies). In immunocompetent individuals CMV infection is rare, usually asymptomatic, and not associated with severe forms of the disease [1,2].

However, CMV colitis in presumed immunocompetent patients is becoming an increasingly recognizable entity with an increase of reported cases in the last few years and multiple studies pointing out some risk factors such as advanced age (above 55 years), diabetes mellitus, chronic renal failure, pregnancy, steroid use, and red blood cell transfusion, based on the premise that these conditions affect, in one way or another, the immune system [3-7].

The diagnosis is based on clinical suspicion and confirmed with CMV-specific immunohistochemistry in tissue biopsies, even though the “owl eye appearance” intranuclear inclusion bodies are specific for CMV infection [8,9]. Early diagnosis and proper management are essential and impact the prognosis.

## Case presentation

A 91-year-old woman with a medical history of depression, hypertension and type 2 diabetes mellitus with good metabolic control presented at the emergency room with a four day history of watery diarrhea, multiple episodes per day, associated with abdominal pain and bloating. At presentation, she was hypotensive (blood pressure 95/56mmHg), pale, dehydrated, with a very painful, distended and tympanized abdomen.

**Figure 1:**
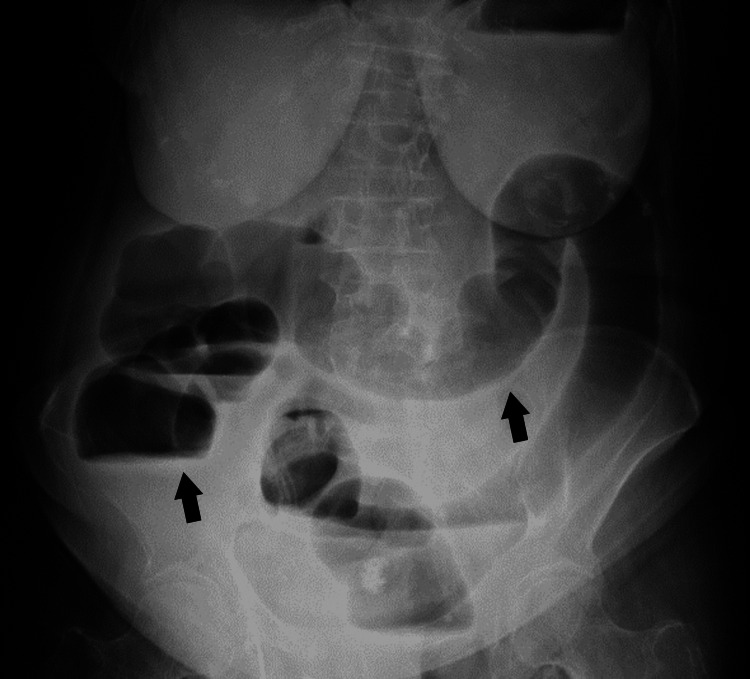
X-ray image of the abdomen X-ray image of the abdomen showing marked dilatation of the entire colon with air-fluid levels (arrows).

**Figure 2:**
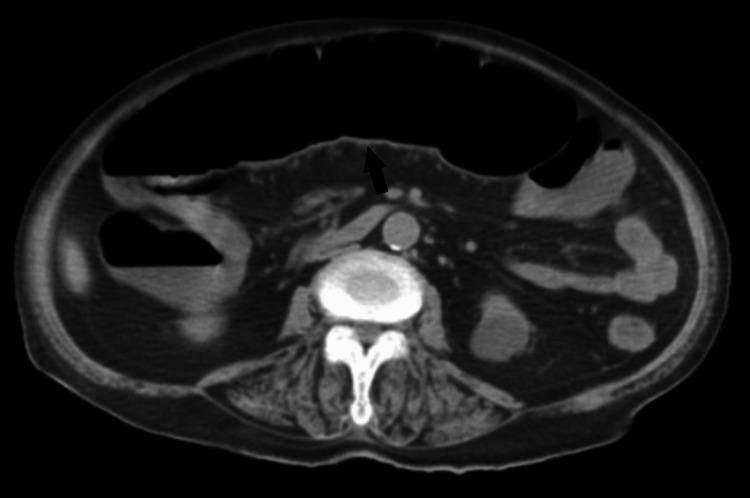
Computer tomography scan of the abdomen and pelvis A transverse cut in the computer tomography scan of the abdomen and pelvis showing a dilated colon with loss of the haustra (arrow).

The initial blood work demonstrated, as displayed in Table 1, mild anemia (hemoglobin 11.6g/dL); elevated inflammatory parameters (leukocytes 12.6x10^9/L, C-reactive protein 22.1mg/dL, ferritin 1403ng/mL); hyperlactacidemia (2.6mmol/L); elevated erythrocyte sedimentation rate (70mm/h); altered kidney function (creatinine 3.17mg/dL, urea 233mg/dL); hypokalemia (3.04mmol/L); hypoalbuminemia (2.5g/dL) and hypoproteinemia (4.9g/dL). Stool studies were negative for Campylobacter, Shigella, Salmonella, Clostridioides difficile and parasites. Abdominal X-ray revealed marked dilatation of the entire colon with air-fluid levels (Figure 1). A computer tomography (CT) scan of the abdomen and pelvis noted findings suggestive of an inflammatory process with colon dilatation, loss of the haustra and air-fluid levels, without any definite obstructive lesion (Figure 2). The patient was hospitalized and empirical antibiotic therapy with piperacillin/tazobactam and metronidazole was initiated.

**Table 1:** Laboratory results

Serum test	Result	Reference range
Hemoglobin (g/dL)	11.6	12.0-15.0
Leukocytes (X10^9^/L)	12.6	4.0-10.0
C-reactive protein (mg/dL)	22.1	< 0.50
Ferritin (ng/mL)	1403	30-340
Lactate (mmol/L)	2.6	0.5-2.0
Erythrocyte sedimentation rate (mm/h)	70	< 35
Creatinine (mg/dL)	3.17	0.50-0.90
Urea (mg/dL)	233	17-49
Potassium (mmol/L)	3.04	3.50-5.10
Albumin (g/dL)	2.50	3.50-5.20
Total serum protein (g/dL)	4.9	6.40-8.30
Hemoglobin A1C (%)	6.6	< 5.7

In the following days after admission, the patient remained very symptomatic, maintaining multiple episodes of loose stools per day associated with a frankly distended and painful abdomen, with minimal or no improvement in the laboratory data and in the abdominal CT-scan images of re-evaluation, despite a full cycle of antibiotic. No surgical indication was established by the general surgeon, therefore, we continued general conservative measures and blood and stool cultures were repeated.

However, two weeks after being hospitalized, the symptoms did not relieve and the patient developed bloody diarrhea, decreasing hemoglobin levels, requiring two units of packed red blood cell transfusion. An abdominal CT scan with contrast was performed, showing no signs of intestinal ischemia as well as no signs of improvement concerning the colonic distension. Stool studies came all negative, once again. The case was again discussed by a multidisciplinary team, and, despite the risks, the patient underwent a colonoscopy examination.

**Figure 3:**
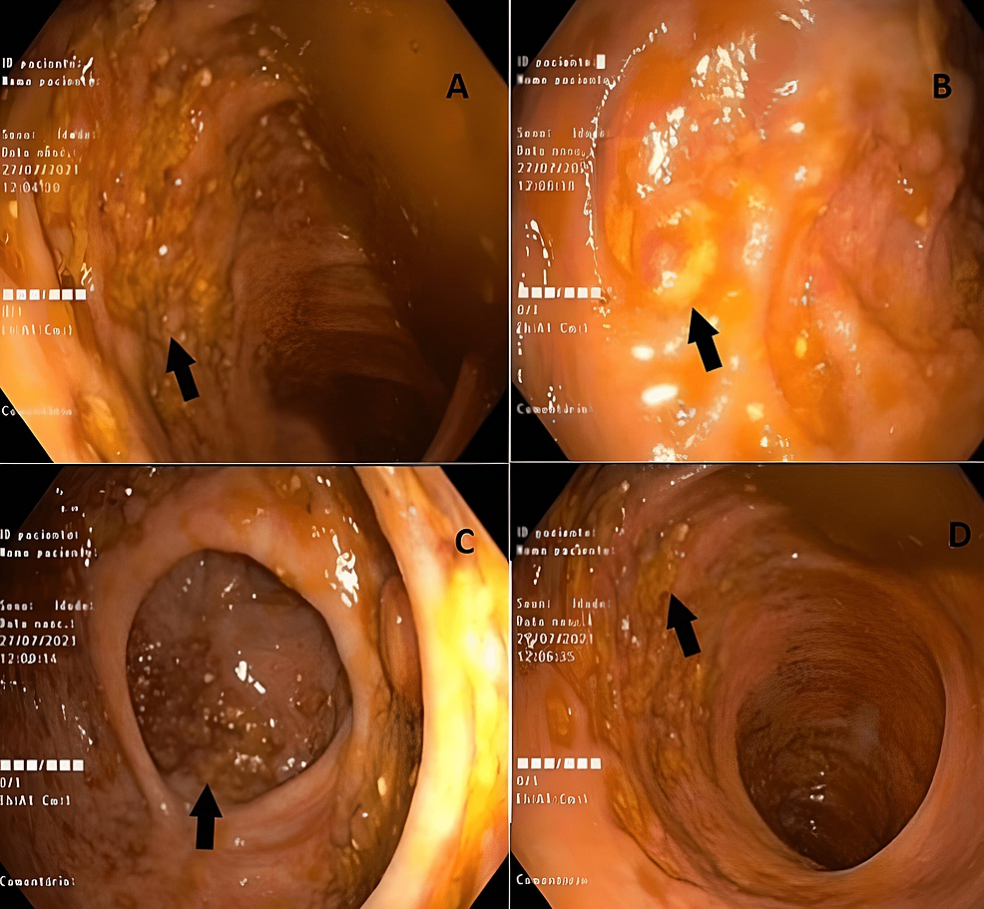
Colonoscopy images 26 days after admission, incomplete colonoscopy was performed, disclosing multiple superficial ulcers in the mucosa (arrows), except in the rectum.

**Figure 4:**
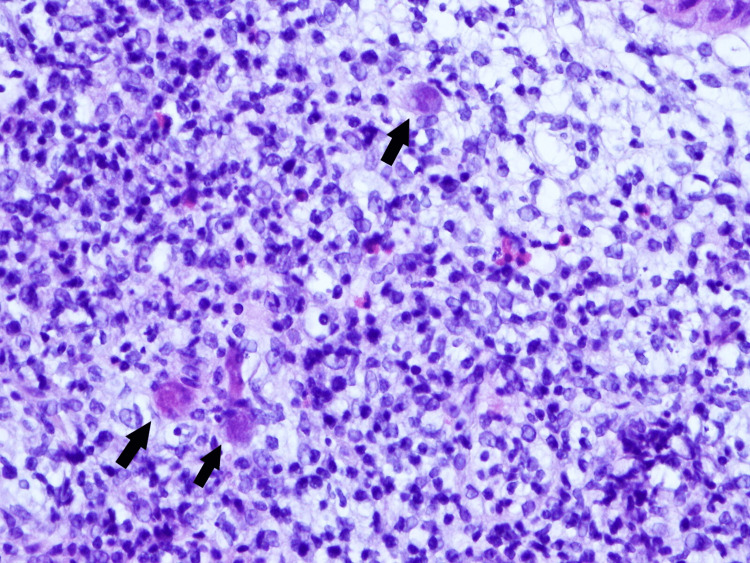
Specimen from colon biopsy with hematoxylin and eosin stain (400x) The large cells with basophilic intranuclear inclusions (arrows) are infected with cytomegalovirus.

**Figure 5:**
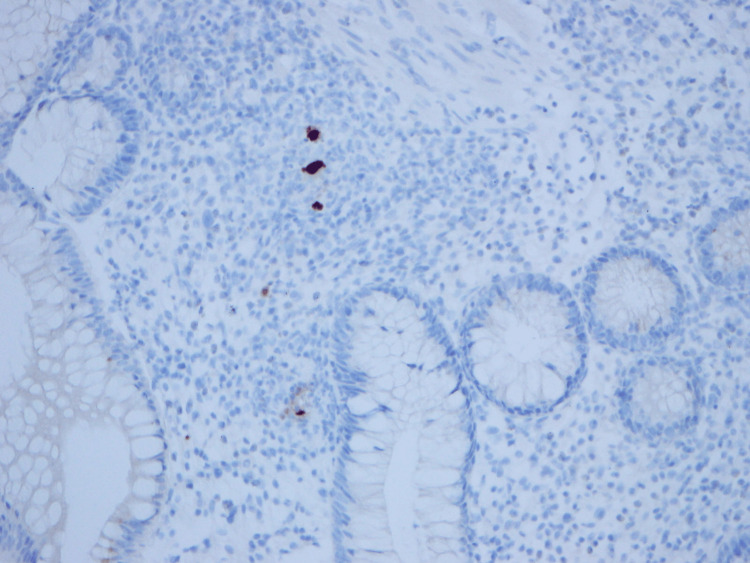
Specimen from colon biopsy with immunohistochemistry specific for cytomegalovirus (40x) The brown cells correspond to the cells infected with cytomegalovirus.

Incomplete colonoscopy was performed, progressing up to 70cm from the anus, disclosing multiple superficial ulcers in the mucosa, except in the rectum (Figure 3). Biopsy result disclosed undergoing colitis with ulcers in relation to a CMV infection (Figures 4, 5). HIV serology was negative and CMV serologies were positive for IgM and IgG antibodies with strong avidity. Under the diagnosis of CMV infection, ganciclovir 5mg/Kg 12/12h was prescribed and the patient's symptoms gradually improved over the next days. There was a significant and sustained decrease in inflammatory parameters and abdominal and pelvis CT-scan images of re-evaluation noted an almost complete resolution of the colonic distension. 

Therefore, the therapy was switched to oral valganciclovir 900mg twice a day and the patient was discharged on August 25th, 54 days after being admitted to the hospital. 

A medical reassessment appointment was scheduled for September 23 to assess the need to continue treatment, however the patient died in the meantime. 

## Discussion

CMV infection is common among immunocompromised individuals. In this set of patients, the spectrum of disease is very wide, ranging from encephalitis to pericarditis, and usually associated with a considerable morbidity and mortality [1,2].

In immunocompetent individuals CMV infection is rarer and usually asymptomatic, with a mononucleosis-like syndrome the most common form of symptomatic disease. However, there has been growing evidence that CMV infection in presumed immunocompetent individuals is not as rare as previously thought [1,2,10].

Gastrointestinal involvement is unusual in this group of patients but when it occurs the colon seems to be the most frequent site affected. A systemic review from 1950 to 2007 identified only 91 immunocompetent patients with gastrointestinal CMV infections and in a meta-analysis performed by Galiatsatos et al. only 44 immunocompetent patients with CMV colitis were identified in a 23-year period [2,6,11-14]. 

Despite being rare, CMV colitis in immunocompetent individuals has been an increasingly recognizable entity with some underlying conditions being pointed out as risk factors, such as advanced age (above 55 years), diabetes mellitus, chronic renal failure, pregnancy, steroid use, and red blood transfusion. Though, the latter two were the only ones reported as independent risk factors for the development of CMV colitis in this set of patients [3-7,11].

A literature review from 2016 to 2021 yielded 20 cases of CMV colitis in immunocompetent hosts with a mean age of 67.45 years (per search using Pubmed indexing keywords). Among those, only six patients had no reported comorbidities, the remaining 14 had underlying conditions, with hypertension (40%); diabetes mellitus (30%) and chronic renal failure (15%) the most frequent. These findings are consistent with other studies such as the extensive literature review made by Karigane et al., where from all the 33 cases of CMV enteritis or colitis identified the mean age was 68 years old and the meta-analysis performed by Galiatsatos et al. where from the all the 44 immunocompetent patients diagnosed with CMV colitis, only 10 had no associated co-morbid conditions [6,8,13,15,16]. Also, a recent study that compared CMV colitis in immunocompromised versus immunocompetent patients found that in the immunocompetent group the patients were older, had more comorbidities and were in worse general condition [17].

Aging seems to be associated with a decline in humoral and cellular immunities, particularly the latter, with multiple studies associating it with reduced T cell counts and function. In a series of 19 patients reviewed by Einbinder and colleagues advanced age (above 55 years) was reported to be a risk factor for CMV colitis. When it comes to co-morbid conditions such as diabetes, literature suggests that is associated with an impaired cytokine response and an impaired function of polymorphonuclear cells [6,7,11,18].

The most common presenting symptoms of CMV colitis are diarrhea, fever, abdominal pain and, in 50% of the cases, hematochezia [6,14,19]. In our case, we had an elderly woman with well-controlled diabetes that presented with abdominal pain and watery diarrhea associated with signs of multiorgan dysfunction. Despite the severity of the case, we did not consider CMV colitis as the most probable diagnosis and based ourselves on the most common options, namely acute bacterial intestinal infection followed by ischemic colitis. We deemed inflammatory bowel disease less probable given the advanced age and the absence of previous history. It is only when the patient developed hematochezia and underwent colonoscopy examination where biopsy specimens were taken that the diagnosis of CMV colitis was made.

This highlights the complexity of the diagnosis of CMV colitis in patients that do not fit the classic definition of immunocompromised. Histology findings are essential for the diagnosis and even though “owl eye appearance” intranuclear inclusion bodies are specific for CMV infection, CMV-specific immunohistochemistry (IHC) is considered the diagnostic gold standard due to its high sensibility to detect CMV in tissue biopsies [8,9].

The current agreement is to use ganciclovir to manage severe forms of CMV disease in immunocompetent patients, however there are limited data when it comes to doses and duration of treatment in this set of patients [14,18-20].

The mortality of immunocompetent patients with CMV colitis is high, particularly in elderly patients with comorbidities, perhaps also due to a delay in the diagnosis and in the initiation of target therapy.

## Conclusions

CMV colitis in immunocompetent individuals is often missed by clinical physicians due to the complexity of the diagnosis in this set of patients. The fact that it is still considered a rare form of the disease and assumed to be a diagnosis only made in patients that fit into the classic definition of immunosuppressed makes the diagnosis so challenging. However, as it was previously mentioned, the number of CMV colitis in immunocompetent individuals reported cases is increasing, particularly in older patients with comorbidities. This should emphasize the need to reframe the definition of an immunosuppressed individual, including those with advanced age and those with underlying conditions that can possibly affect their immune status.

Therefore, CMV colitis should promptly be considered in the differential diagnosis as timely diagnosis and treatment influence the outcome.
